# Feasibility of simultaneous whole-brain imaging on an integrated PET-MRI system using an enhanced 2-point Dixon attenuation correction method

**DOI:** 10.3389/fnins.2014.00434

**Published:** 2015-01-05

**Authors:** Udunna C. Anazodo, Jonathan D. Thiessen, Tracy Ssali, Jonathan Mandel, Matthias Günther, John Butler, William Pavlosky, Frank S. Prato, R. Terry Thompson, Keith S. St. Lawrence

**Affiliations:** ^1^Lawson Health Research InstituteLondon, ON, Canada; ^2^Medical Biophysics, Western UniversityLondon, ON, Canada; ^3^Diagnostic Imaging, St. Joseph's Health CareLondon, ON, Canada; ^4^Fraunhofer Institute for Medical Image Computing MEVISBremen, Germany

**Keywords:** PET-MRI, attenuation correction, arterial spin labeling, ASL, ^18^F-fluorodexoyglucose, FDG, cerebral blood flow, glucose uptake

## Abstract

**Purpose:** To evaluate a potential approach for improved attenuation correction (AC) of PET in simultaneous PET and MRI brain imaging, a straightforward approach that adds bone information missing on Dixon AC was explored.

**Methods:** Bone information derived from individual T1-weighted MRI data using segmentation tools in SPM8, were added to the standard Dixon AC map. Percent relative difference between PET reconstructed with Dixon+bone and with Dixon AC maps were compared across brain regions of 13 oncology patients. The clinical potential of the improved Dixon AC was investigated by comparing relative perfusion (rCBF) measured with arterial spin labeling to relative glucose uptake (rPET_dxbone_) measured simultaneously with ^18^F-flurodexoyglucose in several regions across the brain.

**Results:** A gradual increase in PET signal from center to the edge of the brain was observed in PET reconstructed with Dixon+bone. A 5–20% reduction in regional PET signals were observed in data corrected with standard Dixon AC maps. These regional underestimations of PET were either reduced or removed when Dixon+bone AC was applied. The mean relative correlation coefficient between rCBF and rPET_dxbone_ was *r* = 0.53 (*p* < 0.001). Marked regional variations in rCBF-to-rPET correlation were observed, with the highest associations in the caudate and cingulate and the lowest in limbic structures. All findings were well matched to observations from previous studies conducted with PET data reconstructed with computed tomography derived AC maps.

**Conclusion:** Adding bone information derived from T1-weighted MRI to Dixon AC maps can improve underestimation of PET activity in hybrid PET-MRI neuroimaging.

## Introduction

The development of positron emission tomography (PET) and magnetic resonance imaging (MRI) hybrid systems that provide the combined advantages of high molecular sensitivity of PET and high spatial resolution of MRI, among other benefits, has led to ~400% increase in PET-MRI imaging publications in the last 4 years (Pubmed search “PET/MRI, PET-MRI, PET/MR, or PET-MR”). The clinical potential of this hybrid approach in neuroimaging extends beyond image fusion considering the ability of MRI to collect data associated with not only anatomy but also function, diffusion and metabolite concentrations (Pichler et al., 2010). In dementia, for instance, accuracy of early and differential diagnosis is improved with combined information from volumetric MRI and PET-specific tracers (Morbelli et al., 2010; Dukart et al., 2011), where MRI is not only used for anatomical localization of PET tracer but also to correct misclassification of gray matter voxels (Gutierrez et al., 2012) and to aid in retrospective motion correction of PET data (Catana et al., 2011). In neuro-oncology, metabolically active tumors can be more clearly delineated and better characterized with PET-MRI (Catana et al., 2012). In neuroscience, PET-MRI provides the ability to explore neurophysiologic processes such as neurovascular coupling or neuromodulation within one integrated dynamic model (Catana et al., 2012). These neurological applications are achieved with scanners that offer simultaneous hybrid imaging where diagnostic accuracy is maximized as image registration errors and total image acquisition times are minimized, and physiological and metabolic states are identical.

Accurate correction of PET signal attenuation in PET-MRI hybrid systems using information derived from MRI is challenging. Inherent limitation(s) of various proposed methods can lead to PET quantification errors of up to 20% (Keereman et al., 2013; Andersen et al., 2014; Dickson et al., 2014; Hitz et al., 2014), which limits adoption of PET-MRI in neuroimaging. Commercial PET-MRI hybrid systems rely on MRI-derived attenuation correction (MRAC) using a 2-point Dixon method (Dixon, 1984) based on segmentation of MR signals from water and fat into air, lung, fat and soft tissue (Coombs et al., 1997) but ignores bone, rendering this method less ideal for brain imaging. Various methods have been explored to produce optimal MRAC maps for neuroimaging that are as accurate as clinically accepted AC maps produced by computed tomography (CT). Ultra-short echo (UTE) MRI (Catana et al., 2010) where bone is imaged and incorporated into AC maps, can suffer from long imaging time (~5 times longer than Dixon - too long for whole-body imaging but still feasible for brain imaging), low spatial resolution and inaccurate signal segmentation (Dickson et al., 2014). Methods that employ CT templates/atlases mapped to individual MRI (Beyer et al., 2008; Marshall et al., 2013) or to guide MRI segmentation (Hofmann et al., 2008; Kops et al., 2009; Poynton et al., 2014), require complex algorithms that have limited reproducibility and at best still retain some level of inter-modality misregistration (Pappas et al., 2005), especially where large signal intensity differences exist between MRI and CT (e.g., cortical bone).

In this study, we explored the feasibility of simultaneous measurements of brain function with PET and MRI using an enhanced Dixon-based MRAC for PET signal attenuation correction in an integrated whole-body PET-MRI system. We combined bone segmentation from high-resolution three-dimensional (3D) T1-weighted anatomical images, routinely acquired in brain imaging, with the standard Dixon MRAC method implemented by the manufacturer. Bone segmentation was achieved with segmentation tools available from SPM8 (http://www.fil.ion.ucl.ac.uk). Comparison between the Dixon and enhanced Dixon method was made across a number of brain regions to assess regional differences and performance. In addition, PET ^18^F-fluoro-deoxyglucose (^18^F-FDG) data corrected with the enhanced method were compared to cerebral blood flow (CBF), measured simultaneously with 3D pseudo-continuous arterial spin labeling (pCASL), to investigate the potential clinical application of this MRAC method, for instance, in future neurodegenerative studies where hybrid PET-MRI is highly recommended (Drzezga et al., 2014).

## Materials and methods

### Participants

This study was approved by the University Research Ethics Board, and written informed consent was obtained from all subjects. PET-MRI images were acquired from oncology patients recruited following the completion of a clinical PET-CT exam as part of their healthcare management. A total of 13 oncology patients were recruited 7 males and 6 females (58 ± 8 years old). All subjects had no history of neurological disorder and no radiation therapy or chemotherapy prior to imaging and were referred for whole-body PET-CT exam for staging of oncology. To minimize errors in MRI image quality from potential image artifacts produced by metal implants such as MR signal loss, only patients free of metallic implants and fixtures including dental implants were included in this study.

### PET-MRI image acquisition

Whole-brain PET and MRI data were acquired simultaneously on a Siemens Biograph® mMR system. The PET subsystem consists of 8 rings of 56 detector blocks, each housing 8 × 8 lutetium oxyorthosilicate (LSO) crystals (size = 4 × 4 × 20 mm^3^) coupled to a 3 × 3 array of avalanche photodiodes (APDs) producing a transaxial field of view (FOV) of 59.4 cm and an axial FOV of 25.8 cm. The 3T MRI subsystem is similar to a Siemens 3T Verio with a maximum gradient strength of 45 mT/m and slew rate of 200 T/m/s, but a reduced bore size of 60 cm. For detailed description of the Biograph® mMR specifications cf. Delso et al. (2011). All MR images were acquired using a PET-compatible 16-channel phased array head (12-channel) and neck (4-channel) radiofrequency (RF) coil.

PET-MRI data were acquired immediately following the completion of a 20-min whole-body PET-CT scan and following 60-min uptake of a single intravenous injection of ^18^F-FDG (5 MBq/kg) administered in a dimly lit room. All subjects fasted for a minimum of 6 h and had blood glucose levels between 4.8 and 5.8 mmol/L. Clinical whole-body PET-CT were acquired following standard oncology imaging protocol from the level of the eyes to the mid-thigh. A series of MR images were acquired during a 15 min PET list-mode acquisition with subjects in supine position. The MRI data included:

- Coronal T1-weighted dual-echo 3D VIBE-Dixon for MRI Dixon-based attenuation correction of PET data [repetition time (TR) = 3.60 ms, echo times (TE) = 1.23 and 2.46 ms, flip angle = 10°, FOV = 500 × 328 mm^2^, 128 slices, voxel size = 4.1 × 2.6 × 3.1 mm^3^, bandwidth = 965 Hz/Px, acceleration factor of 2, and total acquisition (TA) = 0.19 s] (Martinez-Möller et al., 2009).
- Sagittal T1-weighted 3D magnetization-prepared rapid gradient-echo (MPRAGE) sequence (TR/TE: 2000/2.98 ms, inversion time (TI) = 900 ms, flip angle = 9°, FOV = 256 × 256 mm^2^, 176 slices, isotropic voxel size = 1.0 mm^3^, bandwidth = 240 Hz/Px, an acceleration factor of 2 and TA = 4.48 min).
- Transverse 3D single shot gradient-and-spin-echo (GRASE) pCASL sequence (Günther et al., 2005) (TR/TE = 3500/22.76 ms, FOV = 240 × 240 mm^2^, 24 slices, voxel size = 3.8 × 3.8 × 5 mm^3^, bandwidth = 2298 Hz/Px, and acceleration factor of 2). The pCASL label consisted of 1.5 s train of RF pulses applied 9 cm below the center of the imaging volume with a mean gradient of 0.6 mT/m. Sixty-four label and control pairs were acquired after a post-label delay of 1.2 s for TA = 7 min. Two nonselective inversion pulses were applied for background suppression during the post-labeling delay. Proton density images (M_0_) were acquired with the pCASL sequences for CBF quantification using a TR of 5 s, with no labeling or background suppression pulses.

### MR attenuation map (μ-map) generation and PET image reconstruction

Two MRAC segmentation methods were used for attenuation correction of the PET data: the standard 2-point Dixon method (Martinez-Möller et al., 2009) and 2-point Dixon method plus bone segmentation (Dixon+bone), which is described below. The Dixon MRAC μmaps were generated online from segmentation of fat and water signals in the in- and out-of-phase Dixon images into fat, soft tissue and air/background, as implemented by the manufacturer. Linear attenuation coefficients of 0, 0.086, and 0.10 cm^−1^ were assigned to air, fat and soft tissue, respectively (Martinez-Möller et al., 2009). Attenuation correction factors for hardware including the RF coil and scanning bed were included in the Dixon μ-map. The Dixon+bone MRAC μ-maps were generated offline using SPM8 and in-house MATLAB (2012a, The MathWorks, Natick, MA) scripts. This method overlays a bone mask created from bone segmentation of the MPRAGE image to a Dixon μ-map and applies a linear attenuation coefficient (μ_a_) of 0.143 cm^−1^ (Catana et al., 2010), the μ_a_ value of bone at 511 kev, to the bone mask.

The bone mask for each subject was created as follows. (1) The T1-weighted MRPAGE dataset was coregistered to the Dixon in-phase image in SPM8 using rigid-body transformation with a normalized mutual information cost function to place the MPRAGE in the same voxel space as the Dixon μ-map. (2) The coregistered MPRAGE image set was segmented into gray matter, white matter, cerebrospinal fluid, bone, soft tissue and air/background probability tissue maps in native space using the new segment function in SPM8 and ICBM Tissue Probabilistic Atlases (http://www.loni.usc.edu/ICBM/). The new segment toolbox employs the unified segmentation method (Ashburner and Friston, 2005) that combines affine registration to ICBM atlas, bias field correction and segmentation within one integrated model. (3) The bone probability map was down-sampled to the Dixon images matrix size, smoothed with a 4 mm Gaussian filter (Wagenknecht et al., 2011) and thresholded for probabilities above 80% to minimize inclusion of non-bone signals. (4) The resulting binary image was eroded with morphological filtering and connected component analysis using a 3 × 3 × 3 voxel size of ones before assigning μ_a_ of 0.143 cm^−1^. Erosion was performed to minimize inclusion of non-bone voxels and the size of the structure elements were determined after empirical evaluation of bone voxels within a region known to have true bone content.

Each subject's PET list-mode data were reconstructed using the Siemens e7 tools to one image volume 344 × 344 × 127 matrix with an iterative algorithm (ordered-subsets expectation maximization: 3 iterations, 21 subsets; 3D Gaussian filter with a full width half maximum (FWHM) = 2.0 mm and 2.5 zoom factor) and corrected for decay, dead time, scatter and attenuation. The reconstruction was performed twice using either the Dixon (PET_dx_) or the Dixon+bone (PET_dxbone)_MRAC μ-map.

### Image analysis

To allow for group comparison between PET_dx_ and PET_dxbone_, the PET images were spatially normalized to the Montreal Neurological Institute (MNI) PET template in SPM8 (http://www.fil.ion.ucl.ac.uk) using an affine transformation and non-linear warps, and smoothed with a 6-mm FWHM Gaussian filter. The gray matter (GM) probability map for each subject was spatially normalized to the T1 MNI template using the unified segment approach in SPM, also smoothed with a 6-mm Gaussian filter, transformed to a binary mask at a threshold of 80% and applied to the PET_dx_ and PET_dxbone_ images.

The difference between the Dixon+bone and the Dixon MRAC method across the brain was computed as the percent relative difference (% RD) in mean activity concentration (Bq/ml) in thirteen a priori regions of interest (ROIs). $\%RD=\left[\frac{\left(PET_{dxbone}-PET_{dx}\right)}{PET_{dx}}\right]\;\times \;100$. ROI brain masks were created using the automated anatomical library (Tzourio-Mazoyer et al., 2002) incorporated within WFU PickAtlas toolbox version 3.0 (Maldjian et al., 2003). The ROIs were frontal lobe, cingulate gyrus, insula, parietal lobe, temporal lobe, hippocampus, amygdala, thalamus, caudate, globus pallidus, putamen, and cerebellum. The PET_dx_ and PET_dxbone_ images were scaled by their respective global gray matter mean value to allow for voxel-by-voxel comparison between glucose uptake and blood flow, and since absolute quantification of PET-FDG with tracer kinetic modeling was not feasible in this study and generally not feasible in clinical practice. Regional %RD in relative mean activity was also calculated in the scaled PET data (rPET). To investigate potential regional differences, the mean values extracted from individual ROIs were compared between the two PET data sets using paired *t*-tests with SPSS 20.0 statistical software (IBM Corp. Armonk, NY, USA). Statistical significant differences were set at threshold *p* < 0.05. Evaluation of the regional differences between PET_dx_/PET_dxbone_ and a ground truth method such as PET-CT were not made (Supplementary Material illustrates the comparison in one subject).

pCASL preprocessing was performed with SPM8 and scripts written in MATLAB. The pCASL time series were motion corrected, pair-wise subtracted, and time-averaged. The mean signal was registered to the MPRAGE images using a rigid-body transformation and converted to CBF using a single-compartment flow model (Wang et al., 2003). The CBF images were spatially normalized to the MNI template and smoothed using a Gaussian filter with a FWHM of 6 mm. A GM mask was applied to the CBF images that were normalized by the global mean. To determine the temporal signal-to-noise ratio (tSNR) of the perfusion-weighted time series when acquired with a PET-compatible head coil, tSNR was calculated for each subject as defined by the mean pixel signal in the whole brain relative to the mean pixel standard deviation.

Whole brain voxel-by-voxel independent samples *t*-tests restricted within a GM mask were performed on the individual relative images in SPM8 to investigate differences between rCBF and rPET images. Statistical significant differences were identified for clusters greater than 50 voxels at *p* < 0.05 after correcting for multiple comparisons using the False Discovery Rate (FDR). Voxel-by-voxel Pearson correlation was conducted on relative images averaged across subjects within the thirteen ROIs to correlate rCBF to rPET_dx_ and to rPET_dxbone_.

## Results

### Study participants

A full description of the study demographics is listed in Table 1, including age, gender and diagnosis. No neurological lesions or gross neuropathological abnormalities were observed on PET-MRI brain images. Images were reviewed by a Board Certified Radiologist and Nuclear Medicine physician. Mean and standard deviation of the time from ^18^F-FDG injection to PET-MR examinations were 103 ± 11.18 min.

**Table 1:** **Study demographics**.

**Number**	**Age (years)**	**Gender**	**Primary oncology disease**	**Net injected dose (MBq)**
1	62	F	SPN	368
2	55	M	SPN	390
3	39	M	Germ cell (Testicular)	500
4	64	M	SPN	422
5	62	F	SPN	288
6	62	F	SPN	229
7	54	M	SPN	437
8	51	F	SPN	200
9	56	M	SPN	418
10	65	F	Colorectal	337
11	61	F	NSCLC	411
12	71	M	SPN	388
13	53	M	NSCLC	396

*SPN, Solitary Pulmonary Nodule; NSCLC, non-Small cell lung cancer; M, Male; F, Female*.

### Evaluation of MRAC methods

**Figure 1:**
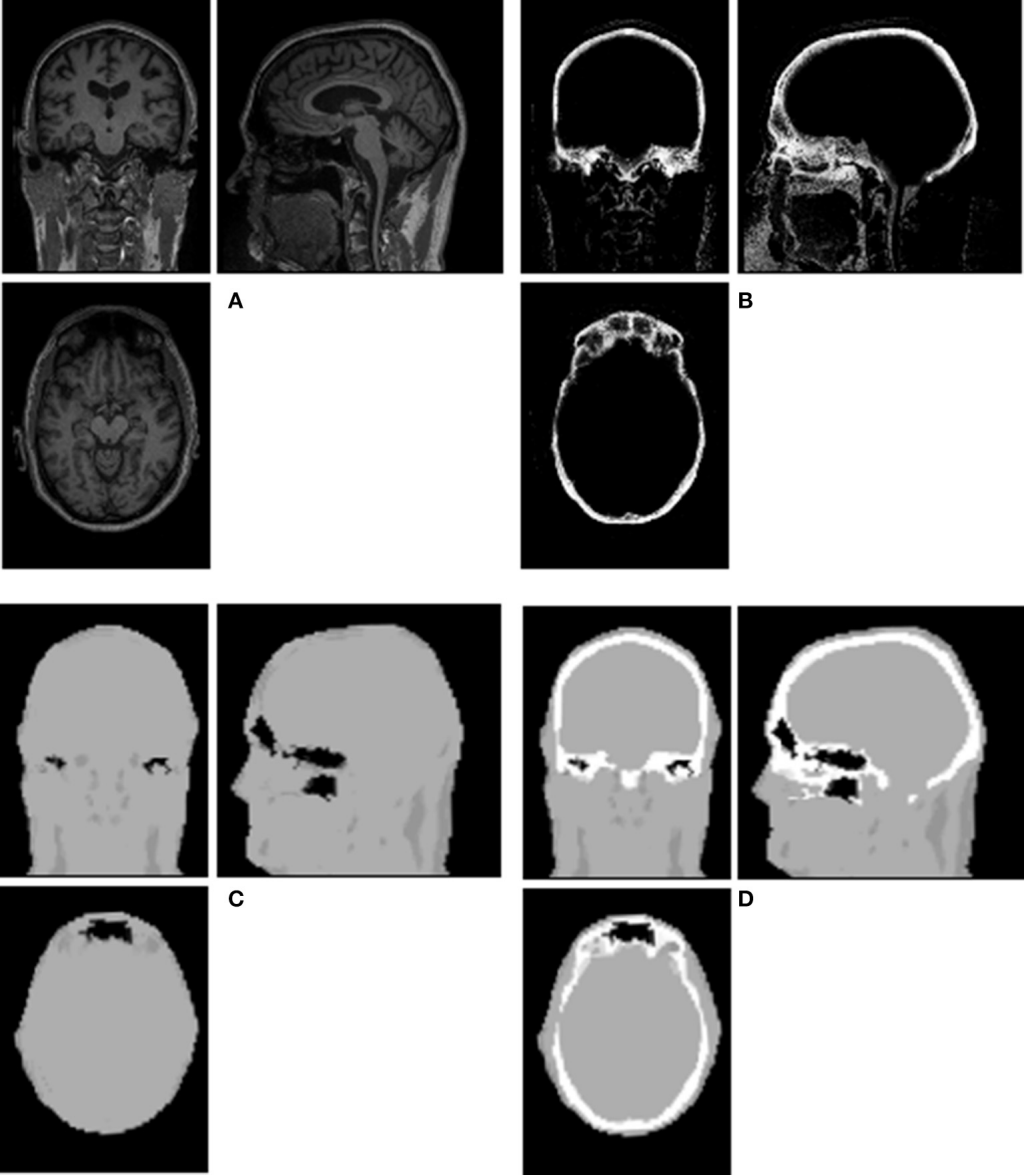
**Illustration of a Dixon+bone μ-map generated in a representative subject**. Images **(A)** T1-weighted MPRAGE, **(B)** bone probability map, **(C)** Dixon μ-map, and **(D)** Dixon+bone μ-map are shown in axial, coronal and sagittal views of a single slice.

**Figure 2:**
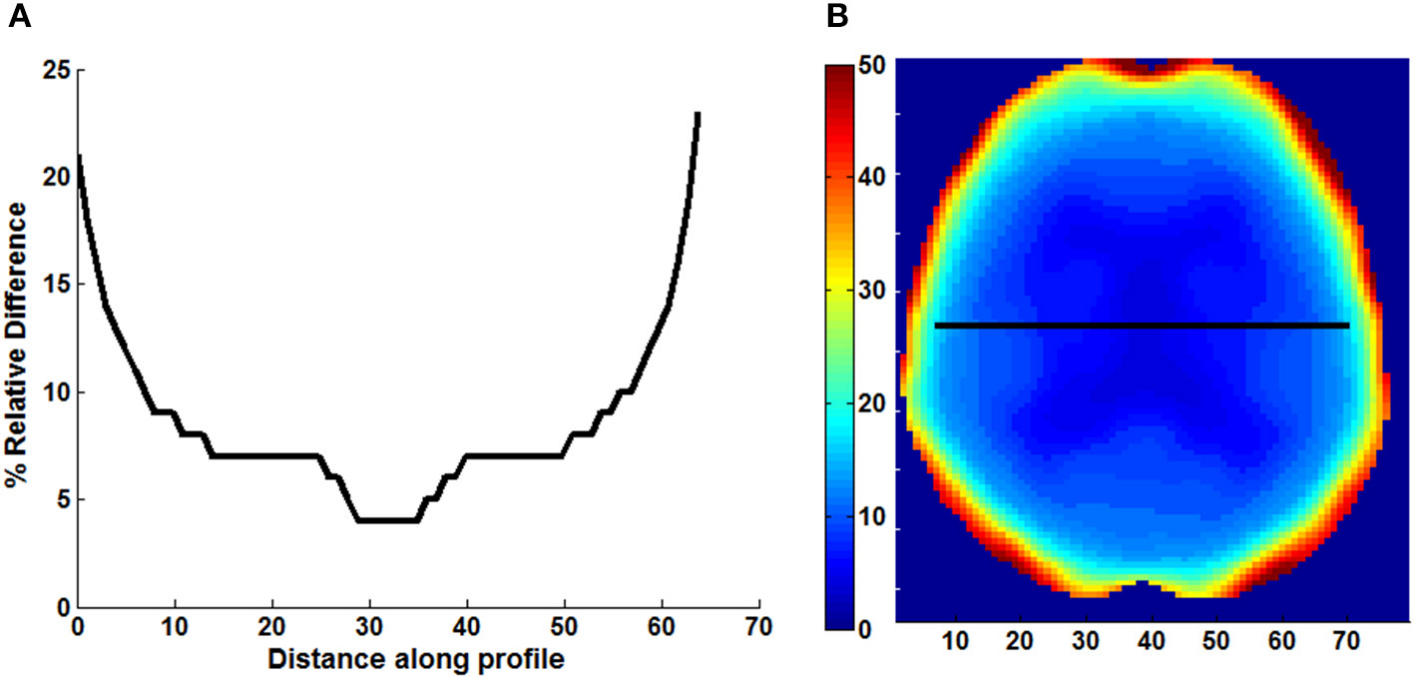
**Line profile (A) across the center slice of a map (B) of relative difference (% RD) between PET images reconstructed with Dixon and Dixon+bone μ-maps**. Images are presented from a representative subject.

**Figure 3:**
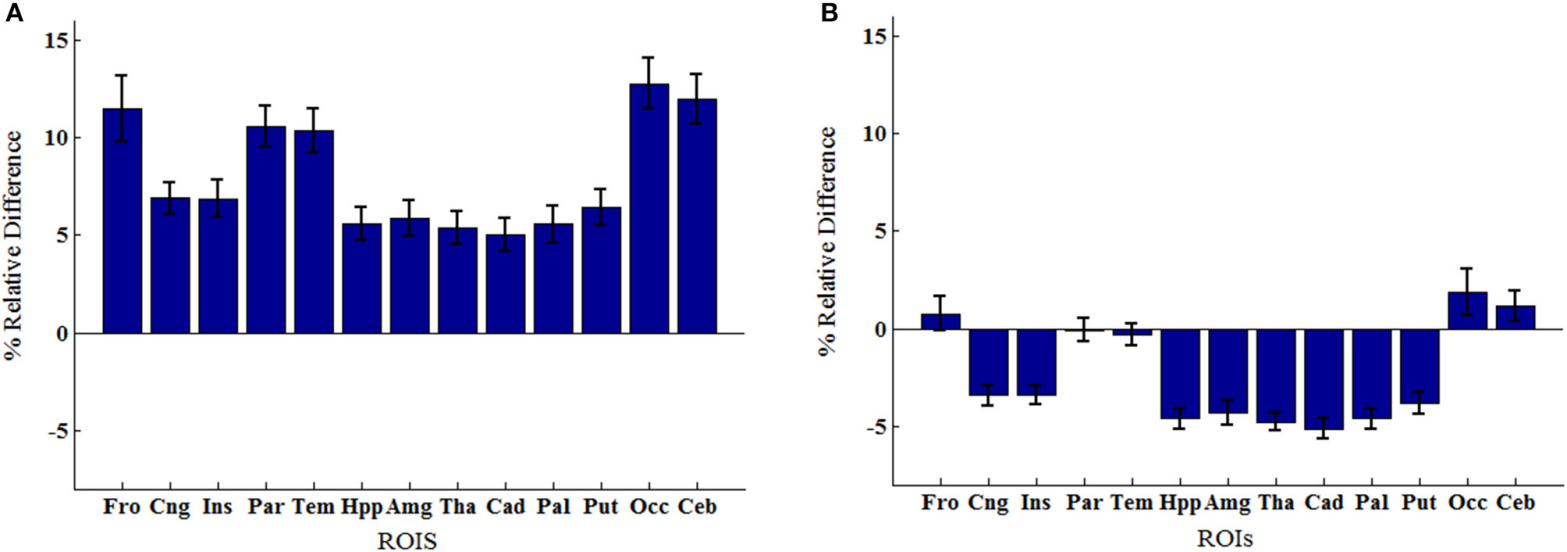
**Regions of interest group means percent relative difference (% RD) in (A) mean activity concentration and (B) mean relative activity between PET signals reconstructed with Dixon and Dixon+bone μ-maps**. Errors bars represent standard deviation on the means. Regions abbreviations: Fro, Frontal; Cng, Cingulate; Ins, Insula; Par, Parietal; Tem, Temporal; Hpp, Hippocampus; Amg, Amygdala; Tha, Thalamus; Cad, Caudate; Pal, Globus Pallidus; Put, Putamen; Occ, Occipital; Ceb, Cerebellum.

An example of Dixon+bone μ-map created by adding bone to a Dixon μ-map from a representative subject is displayed in Figure 1. A gradual radial increase in relative difference between PET images reconstructed with Dixon+bone μ-map compared to Dixon μ-map were observed in all subjects. Figure 2A, illustrates the typical line profile (Figure 2B) through the center slice of the brain of one subject when the PET_dx_ and PET_dxbone_ images are compared. An increase of 5% in the center to nearly 20% in the areas around the cortex was seen. Group mean values and standard deviation of absolute and relative activity in PET_dx_ and PET_dxbone_ for whole brain and regions of interest are listed in Table 2. Paired student *t*-test showed that the absolute mean activity in PET data reconstructed with Dixon+bone μ-map was statistically higher than PET reconstruction with Dixon μ-map in all thirteen ROIs. Conversely, lower relative activity was found in rPET_dxbone_ when compared to rPET_dx_ in all ROIs except, occipital and cerebellum. These regional differences are depicted in Figure 3A as the percent relative difference in mean activity between PET_dx_ and PET_dxbone_, and in Figure 3B as the percent relative difference in rPET_dx_ and rPET_dxbone_ for all thirteen ROIs.

**Table 2:** **Group mean and standard deviation (std) of absolute and relative PET_dx_ and PET_dxbone_ activity concentration (kBq/ml) in regions of interest across the brain**.

**Region**	**Absolute mean activity**					**Relative mean activity**				
	**PET_dx_**		**PET_dxbone_**		***t***	**PET_dx_**		**PET_dxbone_**		***t***
	**Mean**	**Std**	**Mean**	**Std**		**Mean**	**Std**	**Mean**	**Std**	
Frontal	12.70	2.60	14.12	2.86	15.21	0.96	0.04	0.97	0.03	2.95^*^
Cingulate	13.90	2.88	14.88	3.04	17.74	1.06	0.05	1.02	0.05	−22.66
Insula	13.47	2.35	14.39	2.50	17.08	1.03	0.04	0.99	0.04	−24.74
Parietal	13.49	2.71	14.91	2.97	16.99	1.03	0.03	1.02	0.03	−0.74^*^
Temporal	11.63	2.26	12.84	2.51	15.11	0.88	0.02	0.88	0.02	−2.01^*^
Hippocampus	10.35	1.85	10.93	1.97	14.21	0.79	0.05	0.75	0.05	−32.13
Amygdala	10.42	1.87	11.03	2.01	13.53	0.80	0.07	0.76	0.07	−26.43
Thalamus	14.11	2.77	14.87	2.92	13.91	1.07	0.06	1.02	0.06	−34.88
Caduate	11.33	3.10	11.90	3.24	12.47	0.85	0.09	0.81	0.09	−29.34
Pallidum	14.23	2.66	15.02	2.83	12.93	1.08	0.07	1.03	0.07	−36.10
Putamen	16.83	3.21	17.91	3.43	14.21	1.28	0.08	1.23	0.08	−31.08
Occipital	13.18	2.63	14.87	2.95	16.90	1.00	0.06	1.02	0.07	5.74
Cerebellum	10.68	1.78	11.97	2.03	16.46	0.82	0.08	0.83	0.08	5.36
Whole brain	13.16	2.56	14.56	2.81	17.82	1.04	0.10	1.07	0.10	3.05^*^

Results of paired differences between PET_dx_ and PET_dxbone_ are presented as value of the t-statistic (t), where all regional differences met the statistical threshold of p < 0.05 except for

* *where p >0.05*.

### Correlations of perfusion to glucose uptake

**Figure 4:**
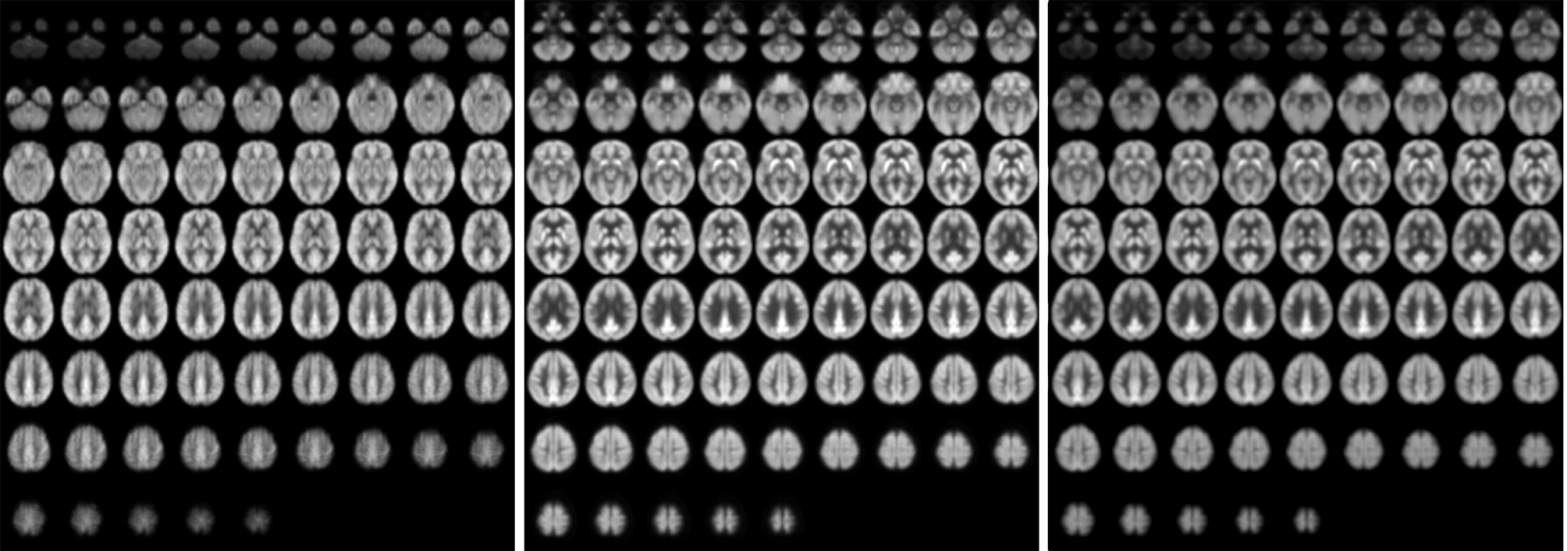
**Whole-brain group maps of relative perfusion and relative glucose uptake from; left to right: pCASL-CBF, ^18^F-FDG-PET reconstructed with Dixon+bone MRAC and ^18^F-FDG-PET reconstructed with Dixon MRAC**. Spatial blurring seen in the bottom row images of the perfusion maps could be minimized with segmented multi-shot 3D GRASE acquisitions.

Images of relative CBF, PET_dx_, and PET_dxbone_ averaged across all subjects are displayed in Figure 4. In general, these group-wise images are similar in appearance. On visual inspection, the group-averaged PET data reconstructed with Dixon+bone μ-map had an apparent higher intensity compared to PET data reconstructed with Dixon μ-map, particularly around the cortex. An additional difference between the data sets was the aliasing artifact observed in the CBF images, which are most visible in distal images. The mean group gray matter CBF was 43.82 ± 4.46 ml/100 g/min. The mean tSNR of whole brain pCASL signal in gray matter across all subjects was 2.83 ± 1.18.

**Figure 5:**
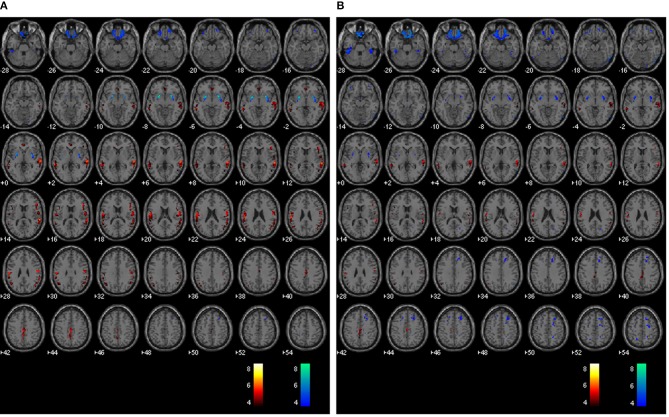
**Whole brain voxel-by-voxel comparison between relative cerebral blood flow and relative ^18^F-FDG-PET activity reconstructed with Dixon (A) and Dixon+bone (B) μ-maps**. Areas with greater relative perfusion are shown in red while areas with greater relative glucose uptake are shown in blue. Statistical differences are set at threshold for cluster >50 voxels, *p* < 0.05 corrected for multiple comparisons with FDR.

The average voxel-by-voxel correlation coefficient across whole-brain gray matter was *r* = 0.63 (*p* < 0.001) between rCBF and rPET_*dx*_, and *r* = 0.53 (*p* < 0.001) between rCBF and rPET_dxbone_. The correlation coefficient for each ROI, derived from voxel-wise comparison between rCBF and rPET_dx_ and between rCBF and rPET_dxbone_, are presented in Table 3 along with corresponding total number of voxels. For the comparison between rCBF and rPET_dx_, moderate-to-high correlations were observed in all ROIs with the highest in the caudate and the lowest in the deep-lying structures of the limbic system such as the hippocampus and amygdala. Similar trends were observed between rCBF and rPET_dxbone_. Results of voxel-by-voxel independent samples *t*-test listed on Table 4, showed areas of significant increase or decrease in relative perfusion compared to relative ^18^F-FDG-PET signals reconstructed with Dixon and with Dixon+bone μ-maps for clusters that met the set statistical threshold. Maps of statistical differences between rCBF and either rPET_dx_ or PET_dxbone_ overlaid on axial slices of a single subject T1-weighted image are displayed in Figure 5. Regions with higher relative perfusion are marked in red while regions with higher relative glucose uptake are marked in blue.

**Table 3:** **Results of voxel-by-voxel Pearson product moment correlation within thirteen regions of interest (*p* < 0.001)**.

**ROI**	**k_e_**	**rCBF vs. rPET_dx_**	**rCBF vs. rPET_dxbone_**	**rCBF vs. PET-CT (Cha et al., 2013)**
Frontal	70169	0.61	0.52	0.41
Cingulate	7598	0.77	0.76	0.61
Insula	3541	0.54	0.53	0.63
Parietal	26845	0.63	0.58	0.5
Temporal	32962	0.72	0.63	0.67
Hippocampus	1797	0.43	0.41	−0.26
Amygdala	414	0.42	0.40	0.087
Thalamus	2149	0.68	0.68	0.45
Caudate	1895	0.85	0.85	0.78
Pallidum	573	0.41	0.41	Not applicable
Putamen	2009	0.48	0.46	0.81
Occipital	21333	0.82	0.73	0.12
Cerebellum	21838	0.54	0.46	0.33

*Correlation coefficients (r) are listed for comparisons between relative ASL-CBF and relative PET-FDG measurements corrected with Dixon and Dixon+bone μ-maps, and comparisons from a previous study using 2D-pCASL sequence and PET-CT. Total number of voxels (k_e_) within each ROI for the MRAC comparisons are included*.

**Table 4:** **Results of independent samples *t*-test in gray matter comparing rCBF to rPET_*dx*_ and comparing rCBF to rPET_dxbone_ for clusters >50 voxels (*p* < 0.05, FDR). MNI coordinates (x–z) and corresponding anatomical location are included**.

**Cluster number**	**Cluster size**	***x***	***y***	***z***	***t*-value**	**Anatomical label**
**rPET_dx_ > rCBF**						
1	431	−22	12	−8	8.67	Left putamen
2	435	28	8	−6	7.84	Right putamen
3	1591	16	34	−28	5.38	Right orbital frontal
4	457	66	−52	−18	4.88	Right inferior temporal
5	569	26	−14	68	4.26	Right precentral
6	685	−20	−54	68	4.25	Left post-central
7	126	−44	−24	−32	4.23	Left inferior temporal
8	61	22	−90	−16	4.16	Right occipital
9	126	20	−54	70	3.71	Right post−central
**rPET_dx_ < rCBF**						
1	1773	0	−22	42	6.91	Left paracentral
2	7935	62	−34	10	6.90	Right superior temporal
3	6368	−56	−28	24	6.24	Left inferior parietal
4	947	−4	42	−4	5.73	Left anterior cingulate
5	268	4	−74	0	4.92	Right lingual
6	59	−2	−86	14	4.55	Left cuneus
7	77	14	−28	6	3.59	Right thalamus
**rPET_dxbone_ > rCBF**						
1	650	26	8	−6	7.9	Right putamen
		−24	6	−6	7.8	Left putamen
2	1591	16	34	−28	6.10	Right orbital frontal
3	1332	66	−52	−18	5.71	Right inferior temporal
		66	−58	6	5.63	Right middle temporal
4	4373	26	−14	68	5.25	Right precentral
5	2848	−20	−36	58	5.05	Left post−central
6	65	−18	−38	38	4.21	Left cingulate
7	79	26	−16	−32	4.09	Right parahippocampal
8	83	4	−34	−12	3.77	Right anterior cingulate
9	70	20	−6	20	3.61	Right caudate
10	76	−20	−72	16	3.58	Left precuneus
**rPET_dxbone_ < rCBF**						
1	1318	62	−34	12	6.14	Right superior temporal
		62	−28	2	5.52	Right middle temporal
2	630	0	−22	44	5.91	Left paracentral
3	668	−62	−38	48	5.16	Left middle temporal
4	238	48	10	18	4.88	Right inferior frontal
5	210	−48	−60	34	4.79	Left angular
6	72	4	−72	18	4.57	Right cuneus
7	73	−46	30	4	4.25	Left inferior frontal
8	82	−44	10	18	4.15	Left insula
9	186	6	42	−8	3.87	Right medial frontal

## Discussion

The feasibility of an integrated whole-body PET-MRI system in simultaneous acquisition of perfusion MRI and metabolic PET neuroimaging with an enhanced attenuation correction method was investigated in this study. Following efforts by other groups to minimize errors in PET signal attenuation corrected with MRI (Kops et al., 2009; Catana et al., 2010; Izquierdo-Garcia et al., 2014; Poynton et al., 2014), and given the lack of consensus on acceptable MRAC methods for bone attenuation (Bailey et al., 2013), we explored a practical workaround for MRAC prior to the start of a neurodegenerative study with PET-MRI.

Direct segmentation of high resolution T1-weighted MR data into various tissue classes for MR attenuation correction of PET have been proposed by Zaidi et al. (2003) and Wagenknecht et al. (2009). Segmentation of voxel intensities were performed with sophisticated algorithms that use fuzzy clustering or *a priori* knowledge of tissue location and shape to derive MRAC μ-maps with ~6% maximum absolute relative difference compared to ground truth (Zaidi et al., 2003; Wagenknecht et al., 2011). However, these methods are time consuming, require post-processing algorithms that are not readily available, and in Zaidi et al.'s (2003) method; require user intervention to improve bone segmentation. In addition, these proposed approaches are sensitive to MR intensity inhomogeneity errors which, when left unaccounted for can exacerbate inherent MR signal issues such as partial volume effects and motion artifacts, potentially inflating bias in MRAC μ-maps. In this study, bone segmentation of T1-weighted data was easily derived with SPM, an automated pipeline routinely used for brain segmentation in neuroimaging. For each voxel, the unified segmentation method in SPM determines intensity distributions of tissue types using a mixed model cluster analysis and spatial priors from a T1-weighted atlas derived from large number of subjects (Ashburner and Friston, 2005). Unlike aforementioned direct segmentation methods, the unified method accounts for non-uniformities in intensity distributions, improving segmentation accuracy (Tsang et al., 2008).

In general, a ~10% increase in whole brain mean ^18^F-FDG activity corrected with Dixon+bone compared to standard Dixon MRAC was observed. A gradual increase in mean ^18^F-FDG activity from ~5% in the center to ~20% in the cortex was observed in PET data corrected with Dixon+bone compared to the same PET data corrected with just Dixon alone, as illustrated in Figure 2 for one individual. This finding was consistent with a recent study comparing PET images corrected with the standard Dixon method to images corrected with Dixon plus bone information derived from individual CT (Andersen et al., 2014). In this study, Andersen et al. (2014) reported a 15% difference in mean activity from the center to the edge of the brain in PET signal reconstructed with Dixon compared to Dixon plus CT bone. Errors caused by not including bone information are more pronounced at the edge than the center of the brain as photons from the edge of the brain travel longer average path lengths through skull than photons from the center (Andersen et al., 2014).

Regional variability in mean activity between PET_dx_ and PET_dxbone_ was also observed in the ROI analysis. Cortical structures such as frontal and temporal lobe had significantly higher relative differences than central brain regions such as basal ganglia and limbic systems (Figure 3A). All thirteen brain regions investigated had statistical significant lower mean activity when reconstructed with the standard Dixon compared to the reconstruction with the Dixon plus bone (Table 2). Similar regional variability in PET-corrected with standard Dixon MRAC compared to CT attenuation maps have recently been observed by other groups (Dickson et al., 2014; Hitz et al., 2014). Scaling the PET images by their global mean removed the overall underestimation but resulted in regional over- and under-estimations (Figure 3B). These regional trends matched results from a previous study (Dickson et al., 2014) and indicate that irrespective of the chosen reference region, cerebellum (Dickson et al., 2014) or global mean as used in the current study, relative PET measurements reconstructed with Dixon still possess MRAC-related bias, albeit slightly reduced. Although signal normalization to a reference value can reduce systemic errors in PET reconstruction, it can inflate regional values particularly in cases where group differences actually exist between the reference values (Borghammer et al., 2008). This might explain the apparent increase in relative PET activity with standard Dixon compared to Dixon+bone (Table 2) or UTE (Dickson et al., 2014) in nearly all regions of the brain. Altogether, the results are in line with previous studies showing that the addition of bone information, either from MRI (UTE/MPRAGE) or CT, to the MRAC maps removes the radial bias (Andersen et al., 2014) and reduces differences in PET signals from ~20 to 10% when compared to the gold standard, CTAC (Kops et al., 2009; Catana et al., 2010; Marshall et al., 2013; Dickson et al., 2014).

The clinical significance of incorporating bone attenuation in PET reconstruction was highlighted by correlating relative cerebral glucose uptake acquired with ^18^F-FDG-PET to relative cerebral perfusion acquired with pCASL across regions in the brain. Recent evidence suggest that a pattern of regional distribution of perfusion to glucose metabolism exist in healthy brains (Newberg et al., 2005; Vaishnavi et al., 2010; Cha et al., 2013) and disruptions to regional blood flow-glucose metabolism coupling are potential markers of brain dysfunction (Vlassenko et al., 2010). In general, a good correlation between perfusion and glucose uptake was observed in this study over the entire brain. Regional variability in relative correlations was found, with the highest correlations in the occipital and caudate, and the lowest in the limbic structures regardless of the MRAC method used (Table 3). However, associations between perfusion and PET signals reconstructed with Dixon+bone closely resemble patterns of regional relative perfusion to glucose metabolism reported elsewhere, with ^18^F-FDG-PET and PET perfusion tracers (Bentourkia et al., 2000; Gur et al., 2009; Musiek et al., 2012), and with ^18^F-FDG-PET and ASL using standalone PET and MRI scanners (Newberg et al., 2005; Chen et al., 2011; Cha et al., 2013).

When compared to Cha et al. (2013), where similar regions were explored with a similar pCASL labeling scheme, higher regional correlations of rCBF to rPET_dxbone_ were observed in the current study, except in the insula and putamen (Table 3). This apparent increase (~30%) in regional coupling of perfusion to glucose metabolism is likely a result of the improved registration accuracy provided by simultaneous imaging and the improved tSNR provided by using a 3D pCASL method (Günther et al., 2005; Vidorreta et al., 2013). The temporal SNR of 3D-GRASE pCASL was not compromised by the use of a PET-compatible head coil, and the mean tSNR reported here was in line with values reported elsewhere (Günther et al., 2005; Vidorreta et al., 2013).

Results from voxel-by-voxel *t*-tests between rCBF and rPET demonstrated regions of hyper- or hypo-perfusion not matched to glucose metabolism (Figure 5 and Table 4). These findings, specifically from PET data corrected with Dixon+bone MRAC, are in line with previous findings of increased resting perfusion to resting metabolism in areas of the brain associated with the default mode network (Gur et al., 2009; Cha et al., 2013), suggesting that these brain regions require increased blood flow due to the sustained state of arousal (Vaishnavi et al., 2010). Apparent bias in bone attenuation from standard Dixon MRAC was evident in the comparison between relative perfusion and glucose uptake as demonstrated in Figure 5 and in comparison to previous studies (Newberg et al., 2005; Chen et al., 2011; Cha et al., 2013). In particular, unexpected increases in rCBF were observed in the left paracentral, left inferior parietal and right superior temporal gyrus when compared to PET_dx_. The spatial pattern of these differences, notably their occurrence closer to the edge of brain, is suggestive of attenuation errors. Indeed, these regions of elevated rCBF were either removed or significantly reduced when compared to PET_dxbone_.

It is possible that segmentation errors such as voxel misclassifications in Dixon+bone μ-maps could account for some differences in correlations between the current study and observations reported elsewhere. Differences in μ_a_ for bone assigned to MR or CT derived μ-maps, and differences in PET scanner geometry and reconstruction parameters including number of iterations, subsets and smoothing kernels could also influence final observations. In addition, limitations inherent to pCASL and to PET-FDG imaging such as partial voluming, differences in cellular localization of blood water and FDG tracers, and optimal selection of post-labeling delay in ASL with minimal compromise to SNR could affect accuracy of regional comparisons (cf. Cha et al., 2013 for detailed description). The evaluation of Dixon+bone μ-maps would have benefited from comparison to ground truth using CT data, which was not feasible in this study. The PET-CT whole body survey acquired on all subjects prior to PET-MRI imaging, permitted coverage solely from the level of the eyes to the thighs as per clinical protocol, limiting whole brain comparisons. As such, absolute and regional performance of the enhanced Dixon μ-maps was not evaluated. Over estimation of bone information was observed in the inferior regions of the brain and can be seen in the Dixon+bone μ-maps (Figure 1 and Supplementary Figures) and in the comparisons of PET_dxbone_ to pCASL CBF (Figure 5). Regions close to the base of the skull where bone anatomy is thin can yield low MR bone signals with MPRAGE (Wagenknecht et al., 2011) or UTE (Dickson et al., 2014) sequences, and these regions are prone to signal misclassification errors in soft tissue/bone/air interfaces in areas around the sinuses- an issue that affects most atlas-based MR-derived attenuation correction methods. Efforts were made to minimize bone misclassification by morphological filtering of non-bone voxels. A recent study demonstrated that SPM8-based method for generating MRAC is robust with a reported accuracy of under 4% when compared to CTAC (Izquierdo-Garcia et al., 2014). The results presented here were in good agreement with previous studies, suggesting that these issues had little or no effect on the study outcome. The regional correlation of pCASL CBF to ^18^F-FDG-PET demonstrated here for the first time with simultaneous imaging can be further improved with imaging of concurrent uptake of the pCASL and FDG tracer.

In general, the agreement between this study and previous studies using CT attenuation maps indicate that Dixon attenuation maps enhanced with bone information from high resolution T1-weighted images can provide a feasible method for correction of PET signal attenuation in PET-MRI neuroimaging. Errors in MRI-derived attenuation correction maps including tissue misclassification can be further minimized by methods that employ tissue classifiers from CT atlas to better guide segmentation of UTE or MPRAGE data (Poynton et al., 2014).

## Author contributions

Conceived and designed the experiments: Udunna C. Anazodo, Keith S. St. Lawrence. Performed the experiments: Udunna C. Anazodo, John Butler. Analyzed the data: Udunna C. Anazodo, Jonathan D. Thiessen, Tracy Ssali, Jonathan Mandel, William Pavlosky, Keith S. St. Lawrence. Interpreted data: Udunna C. Anazodo, Jonathan Mandel, William Pavlosky, Keith S. St. Lawrence. Contributed reagents/materials/analysis tools: Matthias Günther, Frank S. Prato, R. Terry Thompson. Wrote the paper: Udunna C. Anazodo, Keith S. St. Lawrence, Frank S. Prato, R. Terry Thompson.

### Conflict of interest statement

Dr. M. Gunther receives research and development funds from Siemens Healthcare. For the remaining authors none were declared. The authors declare that the research was conducted in the absence of any commercial or financial relationships that could be construed as a potential conflict of interest.
