# Supplementary material for: Feasibility of simultaneous whole-brain imaging on an integrated PET-MRI system using an enhanced 2-point Dixon attenuation correction method
Source: Front Neurosci. 2015 Jan 5;8:434. doi: 10.3389/fnins.2014.00434 (PMC4283546; doi:10.3389/fnins.2014.00434)
Supplement: Supplementary file 1 [file Presentation1.PDF]

Supplementary Material

Feasibility of simultaneous whole-brain imaging on an integrated PET-MRI system using an enhanced 2-point Dixon attenuation correction method.

Udunna C Anazodo*[1,2], Jonathan D Thiessen[1,2], Tracy Ssali[1,2], Jonathan Mandel[3], Matthias Günther[4], John Butler[1], William Pavlosky[3], Frank S Prato[1,2], R Terry Thompson[1,2], Keith S St. Lawrence[1,2].

[1]Lawson Health Research Institute, London, Ontario, Canada.
[2]Medical Biophysics, Western University, London, Ontario, Canada.
[3]Diagnostic Imaging, St Joseph's Health Care, London, Ontario, Canada.
[4]Fraunhofer Institute for Medical Image Computing MEVIS, Bremen, Germany.

Correspondence:
Udunna C Anazodo,
Lawson Health Research Institute,
268 Grosvenor St, London, Ontario, N6A4V2, Canada.
uanazodo@uwo.ca.

1. Supplementary Data

T1-weighted 3D-MPRAGE and T1-weighted dual-echo 3D VIBE-Dixon MR images were acquired on a 52 year old female patient with frontotemporal dementia (FTD) using a Siemens Biograph mMR and imaging parameters described in the methods section.

A retrospective brain CT data acquired a year prior on the same subject was used to compare µ-maps. Axial CT images were acquired using a Philips Brilliance iCT (Philips Healthcare, Cleveland, OH) and following clinical protocol at the London Health Sciences Center, London, Ontario. The imaging parameters include; 512 x 512 matrix size, 30 slices with voxel size of 0.47 x 0.47 x 5mm. No contrast was administered.

Dixon and Dixon+bone µ-maps were generated as described in the methods section. CT images were manually aligned to match the orientation of the MR images and then registered to the Dixon images using SPM8 and a normalized mutual information function. The registered CT images were downsampled to the voxel size of the Dixon images and segmented into bone (300 to 2,000 Hounsfield units [HUs], soft-tissue (-200 to 300 HUs), and air (< -200 HUs). Uniform linear attenuation coefficients were assumed for these 3 tissue classes (i.e., 0.143, 0.100, and 0 cm^{-1}, respectively) as described by Catana et al., 2010. % Relative difference (RD) between the Dixon, Dixon+bone µ-maps and CT µ-maps were calculated as

$$\%RD = \left[\frac{(CTAC - MRAC)}{CTAC}\right] \times 100$$ where CTAC = CT µ-map and MRAC = respective MR µ-maps.

2. Supplementary Figures.

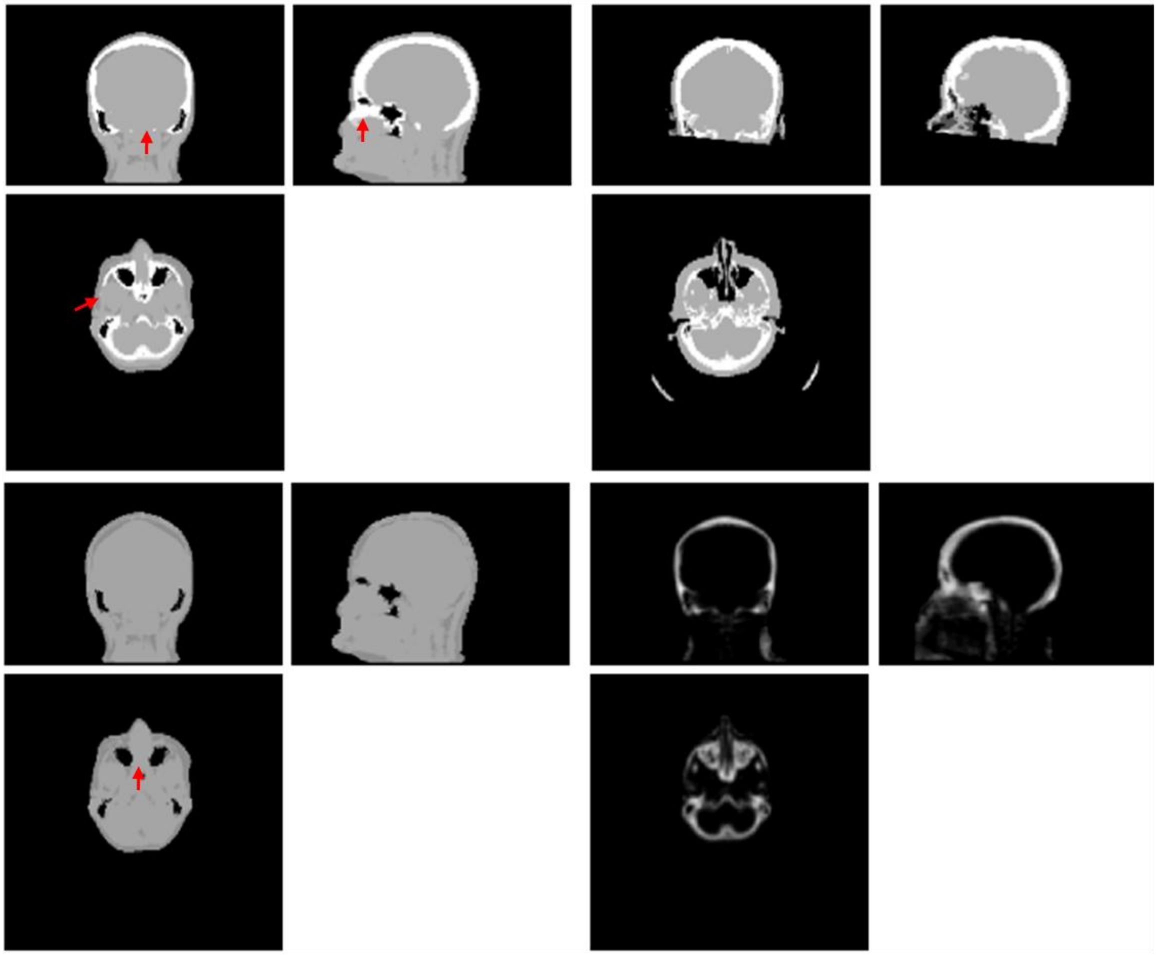

Supplementary Figure I. An illustration of attenuation correction maps generated from the MR and CT images of the FTD patient. Dixon+bone μ-map (top left), CT μ-map (top right), standard Dixon μ-map (bottom left) and corresponding bone segment (bottom right) are shown in coronal, sagittal and axial planes for one slice thorough the lower portion of the brain. Areas of tissue misclassification indicated by the red arrow can be seen in the standard Dixon and Dixon+bone μ-maps.

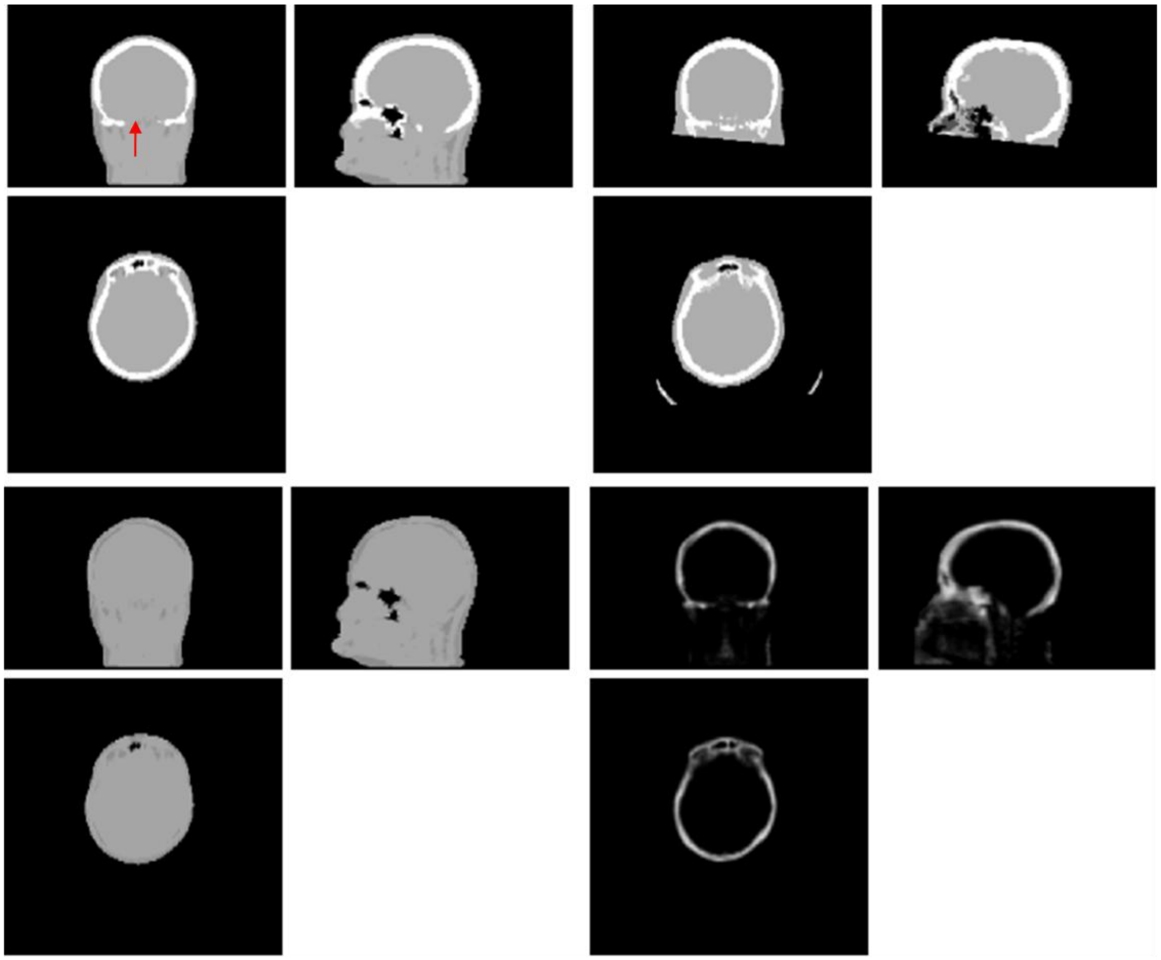

Supplementary Figure II. Another slice through the brain of same subject. Dixon+bone µ-map (top left), CT µ-map (top right), standard Dixon µ-map (bottom left) and corresponding bone segment (bottom right) are shown in coronal, sagittal and axial planes. Areas of missing bone information indicated by the red arrow can be seen in the Dixon+bone.

Figures I and II illustrate a limitation with atlas-based MRAC methods including Dixon+bone. Inferior brain regions close to bone, soft tissue and sinus intersections are more prone to segmentation errors given the poor information from T1-weighted MR images as such errors in bone segments (air/bone) and standard dixon (air/soft tissue) can propagate to the Dixon+bone µ-map generated.

Mean % RD between the CT and Dixon µ-maps and between CT and Dixon+bone µ-maps were -23.92 ± 17.72 % and 8.02 ± 14.95 % respectively. The largest % RD were found in the inferior slices in both µ-maps.

3. References[1]

Catana, C., van der Kouwe, A., Benner, T., Michel, C. J., Hamm, M., Fenchel, M., Fischl, B., Rosen, B., Schmand, M., and Sorensen, a G. (2010). Toward implementing an MRI-based PET attenuation-correction method for neurologic studies on the MR-PET brain prototype. *J. Nucl. Med.* 51, 1431–8. doi:10.2967/jnumed.109.069112.
